# Rational acquisition of laboratory equipment: an accurate mathematical model to estimate the trade-offs in shared and nonshared equipment

**DOI:** 10.1107/S2059798326003608

**Published:** 2026-05-08

**Authors:** C. O. S. Sorzano, J. M. Carazo

**Affiliations:** a National Center for Biotechnology (CNB–CSIC) Madrid Spain; University of Cambridge, United Kingdom

**Keywords:** equipment cost modelling, queueing theory, cryo-electron microscopy, research-facility management

## Abstract

Scientific equipment, such as high-end electron microscopes, entails considerable costs for acquisition, maintenance and operation. Efficient use of these instruments requires thoughtful allocation of access among multiple users. This task may be aided by a thorough statistical analysis that combines cost modelling and queueing theory. In this work, we propose a framework for estimating the hourly access cost and the average waiting time, based on equipment characteristics (price, lifespan and operating hours), user patterns (cycle and acquisition times) and staffing assumptions. In this way, we derive expressions that help estimate the optimal number of users and service congestion levels. The methodology is general and applicable to many types of shared scientific infrastructure. We illustrate its use with realistic cryo-electron microscopy scenarios. To facilitate adoption, we provide an open-access online calculator implementing the model at https://i2pc.es/coss/Programs/equipmentCost.html. This tool can support evidence-based decision-making in equipment planning and policy design.

## Abstract 2

We present a quantitative framework combining cost modelling and queueing theory to estimate hourly access costs, waiting times and congestion levels for shared high-end scientific equipment. The approach is general, illustrated with cryo-electron microscopy examples, and implemented in an open-access online calculator to support evidence-based equipment planning.

## 1. Introduction

Scientific research increasingly relies on sophisticated, high-cost instrumentation that must be shared among multiple users to be economically viable (D’Ippolito & Rüling, 2019 ▸). In structural biology, electron microscopes are a clear example of large equipment that is normally shared: they represent a substantial financial investment, require specialized infrastructure and involve complex operational logistics. At the same time, their value lies in enabling frontier research across fields such as macromolecular biology, biomedical imaging and pharmaceutical discovery.

The question of how to fairly and efficiently allocate access to these instruments, while also justifying the associated investment, has become central for research institutions and funding agencies. We consider two very different ways to approach large-scale equipment acquisition. One is based on a scientific strategy and the other on concrete service considerations. As for the former, an institution may be interested in advancing the growth of a concrete scientific community, attracting top talent or pursuing other strategic reasoning deemed appropriate. We cannot provide statistical support for this process. However, for the latter, for service-based considerations, we can indeed provide statistical analysis combined with queueing theory that considers costs, user patterns and user waiting times (in general, access policies), and is capable of modelling this process, so that key issues such as underutilized or overburdened facilities, inefficient staffing and inequitable resource distribution can be quantitatively addressed from the beginning.

This study proposes a general framework for evaluating the economic and operational efficiency of shared scientific equipment, with the particular example of high-end electron microscopes. The analysis allows estimation of the per-hour access fee, the expected waiting time and the optimal size of the user base under various usage patterns. It considers both the financial cost of the instrument (acquisition, maintenance, fixed and operational costs) and the time cost borne by the researcher (including preparation and analysis time, as well as time spent waiting for access to the instrument).

By quantifying these aspects, we aim to provide institutions and policymakers with tools to:

(i) Assess whether existing equipment is being used efficiently.
(ii) Determine the maximum number of users that can be supported without excessive waiting times.
(iii) Compare centralized versus distributed equipment deployment strategies.
(iv) Design fair and transparent access models that balance scientific opportunity with logistical feasibility.

Although our analysis is illustrated with examples from electron microscopy, the underlying principles apply to a wide range of shared scientific infrastructure, including NMR spectrometers, high-throughput sequencers and beamline-based instrumentation. The goal is not only to reduce costs, but also to enhance the scientific return on national and institutional investments in research infrastructure.

The only similar work that we are aware of is Novikova (2022 ▸), which presents a comprehensive methodology for financial modelling of research infrastructure projects. That work is primarily concerned with evaluating investments at the project and policy level, integrating financial and economic efficiency, redistribution effects, externalities and government support mechanisms. In contrast, our work focuses on the cost of scientific equipment from the end-user or facility manager’s perspective. While we also consider acquisition, operational and fixed costs, our framework is structured around access-based usage: estimating cost per hour of use, understanding shared usage scenarios and analysing waiting times for multiple researchers. Rather than guiding corporate-level procurement decisions, our focus is on resource allocation, cost-sharing and efficiency within scientific facilities, especially in public research institutions.

Despite their centrality to research infrastructure planning, literature addressing these issues remains remarkably scarce. Very few studies, quantitative or qualitative, offer systematic guidance on the economic management of scientific equipment, particularly for high-cost, shared-use instruments. Discussions of laboratory equipment purchases are often relegated to internal administrative processes and rarely appear in academic publications. This absence of analytical frameworks and evidence-based policies is especially notable given the growing importance of cost-efficiency, transparency and sustainability in public research funding. Our work aims to help fill this gap by providing a practical yet rigorous methodology tailored to the real-world needs of scientific institutions and research consortia.

## 2. Methods

### 2.1. Equipment usage cost

We consider a simplified scenario involving *c* pieces of equipment with a lifespan of *L* years (for the examples in this work, we treat the lifespan as the expected time the equipment will be in use, rather than the more typical amortization period). The acquisition cost of each machine is *C*_0_, and its operational cost in the *i*th year is *C*_*i*_. Let *r* denote the annual inflation (or discount) rate, which we will assume to be constant. If there is any space-adaptation cost to host the equipment, it can be added to the acquisition cost *C*_0_.

In this framework, monetary cost is used as an operational proxy for the resources committed to providing access to the machine. The aim is not to equate cost with the total scientific benefit derived from the instrument, since research benefits may also include intangible or nonlinear outcomes such as knowledge generation, talent development, reputational effects or enhanced competitiveness for future funding. Rather, our goal is to estimate the economic cost of using the machine over a given period, regardless of when during its lifespan that usage occurs.

To do so, we first calculate the present value cost (PVC) of the machine, which includes both acquisition and operational expenses discounted to year zero (Pindyck, 1993 ▸),
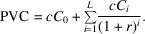


Additionally, we account for fixed costs associated with ownership, such as facility rent, depreciation of the installation area and administrative overhead. Let 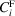 denote the fixed cost incurred in year *i*. The present value of these fixed costs is
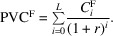


While discounting future costs using the inflation or interest rate *r* is the theoretically correct approach to calculating present value, in practice this treatment can often be simplified without a significant loss of accuracy. In many institutional settings, current values of operational and fixed costs are used directly, based on the understanding that these costs will increase over time due to inflation. Since vendors and service providers typically update their prices to reflect inflation, the nominal increase in future expenses effectively cancels out the discounting applied to bring those costs to present value. As a result, using current cost estimates (for example present-day maintenance or facility rent costs) provides a practical and robust approximation that implicitly accounts for inflation over the lifetime of the equipment. This simplification avoids the need to explicitly carry the interest rate through all cost components while still capturing the long-term financial impact of equipment ownership.

Assuming the machines are expected to operate for a total of *H*^E^ hours per year, the cost per hour of usage, independent of usage timing, is
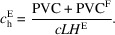


At this point, it is important to distinguish between two conceptually different questions: (i) what is the true access cost per hour of an instrument and (ii) what fraction of that cost is actually recovered from users through access fees. The formulation above addresses the first question. By including the acquisition cost *C*_0_ in the present value cost, we estimate the full economic cost of having an instrument available and operational, regardless of its financing. In many academic settings, the acquisition cost is covered by public funding and is not recovered from internal users, or is only partially recovered from external or industrial users. However, this does not imply that the acquisition cost is negligible or non­existent; rather, it is borne by a different stakeholder (typically funding agencies or governments) at a different point in time. Our focus is therefore on quantifying the access cost, *i.e.* the total cost associated with making the equipment available for use. Decisions about which portion of this cost should be charged to users depend on institutional policies and funding models and can be applied as a subsequent layer on top of the access cost estimated here.

### 2.2. Person cost

The cost per hour of a researcher is derived from the organization’s labour cost, denoted by *S_i_* in year *i*. Assuming that the salary is adjusted annually for inflation, the real cost remains constant over time and equals a fixed value *S*. We denote the hourly cost of a researcher by 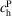. This quantity will be compared with the equipment access fee per hour, 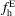
(the computation of which is detailed below). The relative magnitude of these two costs provides a simple criterion for deciding whether to concentrate demand on fewer shared instruments or to acquire additional machines.

### 2.3. Machine usage

Let us assume that the researcher needs to access the equipment on average every *t*_A_ hours of work. This access pattern is modelled as a random process that follows an exponential distribution with mean *t*_A_ hours. The randomness reflects the variability in the researcher’s workflow: depending on the complexity and progress of concurrent tasks, unexpected experimental outcomes or the need to repeat or adjust prior steps, the time between equipment uses may vary significantly.

When accessing the equipment, let us define *t*_acq_ as the acquisition time, *i.e.* the time the researcher spends using the machine, excluding the time needed to prepare for use or analyse the results.

Finally, we define *t*_0_ and *t*_F_ as the time required by the researcher *before* and *after* using the equipment, respectively. These durations account for activities such as sample preparation, setup procedures, data analysis or refractory periods during which the researcher cannot immediately reaccess the machine. While these phases do not involve direct use of equipment, they are essential components of the research workflow and contribute to the overall time and cost structure of each equipment access cycle.

Let us assume that *N*^P^ researchers share access to the same pool of equipment. When the equipment is available, a researcher can use it immediately; otherwise, they must wait in a queue. This scenario is modelled by an M/D/*c*/*N*^P^ queue: M denotes a Markovian arrival process (*i.e.* inter-arrival times are exponentially distributed), D indicates a deterministic service time of duration *t*_acq_, *c* represents the number of identical servers (in this case, the equipment units) and *N*^P^ is the finite number of users generating demand for the resource. The analysis of such systems falls within the field of queuing theory, specifically under the category of *finite source queuing systems* (Sztrik, 2001 ▸). Key results from this theory are summarized below.

We define the average time between two consecutive machine requests by a single researcher as 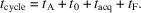
This includes the time before, during and after equipment usage, as well as the stochastic delay *t*_A_ between successive cycles.

The average number of equipment requests per year by a single researcher is then given by 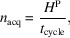
where *H*^P^ is the total number of hours a researcher works per year.

Consequently, the total annual demand for equipment usage time by all researchers is 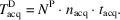


This demand must not exceed the total available equipment time, which is 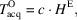
where *c* is the number of equipment units and *H*^E^ is the number of usable hours per year per unit.

### 2.4. Access fee

If we need to buy *c* new pieces of equipment after their usage for *L* years and the full equipment cost (purchase, operating and fixed costs) must be paid from the access fee, 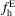, then the access fee must be such that 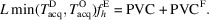
That is, 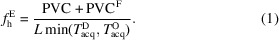
This access fee must be updated every year with inflation, so that in the *i*th year the fee must be 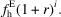


### 2.5. Waiting time in the queue

In the following paragraphs, we will derive the waiting time in the queue based on a birth–death random process.

#### 2.5.1. Birth–death process

Let *n* ∈ {0, 1, …, *N*^P^} denote the number of researchers currently in the system, either using the equipment or waiting. The system evolves as a continuous-time birth–death process with the following rates.

Arrival (birth) rate: 
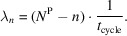
Only idle users (not in the system) can generate new requests.
Service (death) rate: 
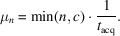
This accounts for up to *c* machines operating in parallel.

#### 2.5.2. Steady-state probabilities

Let 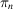 denote the steady-state probability of having *n* users in the system. These probabilities satisfy 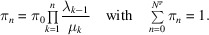
They can be computed recursively and normalized numerically.

#### 2.5.3. Expected waiting time

Let us define the following quantities.

(i) The expected number of users in the system: 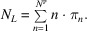
(ii) The expected number of busy machines: 
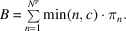
(iii) The expected number of users in the queue: 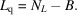
(iv) The system throughput: 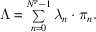
(v) The expected waiting time in the queue (via Little’s law): 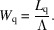

The average waiting time in the queue can be imposed as a service constraint, requiring it to remain below a predefined threshold.

## 3. Examples

Typical labour costs in Spain, including employer contributions, range from 30 000 to 90 000 euro per year. Assuming an annual workload of approximately *H*^P^ = 1800 h, this results in an hourly labour cost of 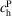 = 16–50 euro h^−1^.

### 3.1. Personal computer

The cost of a personal computer used for standard office tasks is approximately *C*_0_ = 1000 euro, with a typical life span of five years (*L* = 5, even though the accounting amortization time may be shorter). We assume no additional operational or fixed costs, *i.e.*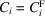 = 0 euro.

Suppose a researcher uses the computer for *t*_acq_ = 3 h per day and requires access every day, *t*_cycle_ = 8. Given a yearly workload of *H*^P^ = 1800 h, this implies *n*_acq_ = (1800/8) = 225 equipment accesses per year. The total annual demand is therefore 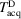 = 22 × 3 = 675 h. The resulting access fee per hour of use for a single researcher is
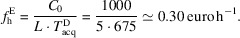


Now consider that the same computer is shared by two researchers, each following the same usage pattern (3 h of use every 8 h of work). If no explicit scheduling is implemented to coordinate their turns, the system behaves like a finite-source queue. In steady state, the computer is

(i) idle 49% of the working hours,
(ii) serving one researcher 37% of the working hours
(iii) and has one researcher being served while the other waits 14% of the working hours.

Under this shared-use configuration, the access fee per researcher drops to
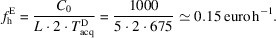


However, sharing the computer introduces an average waiting time of approximately *W*_q_ = 0.8 h per access. Although the access fee is halved under this configuration (from approximately 0.30 to 0.15 euro h^−1^), the cost of the researcher’s time, typically between 16 and 50 euro h^−1^, far exceeds the savings in equipment cost. As a result, it is more cost-effective to assign a dedicated computer to each researcher rather than incur the productivity loss associated with queuing.

### 3.2. Laboratory centrifuge

A laboratory centrifuge typically has a similar cost to a personal computer, approximately *C*_0_ = 1000 euro, but a longer average lifespan of about *L* = 7 years. A structural biologist may require access to the centrifuge approximately three times per working day, corresponding to a cycle time of *t*_cycle_ = 3 h and an acquisition time of *t*_acq_ = 0.08 h (approximately 5 min). This results in an annual equipment usage of 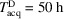. The corresponding access fee for a single dedicated user is
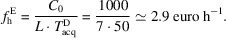


Although this cost is still lower than a typical researcher’s hourly salary, further savings can be achieved by sharing the equipment. For instance, if *N*^P^ = 10 researchers share a single centrifuge, the access fee drops to approximately 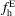 = 0.29 euro h^−1^. The average waiting time in this configuration is modest, around 2 min per access, and is generally considered to be acceptable. For this reason, many laboratories choose to operate with a single centrifuge shared among all researchers.

### 3.3. Screening electron microscope for structural biology

Consider the case of a researcher who accesses a screening electron microscope approximately once every three weeks, corresponding to a cycle time of *t*_cycle_ = 504 h, with each session lasting around *t*_acq _= 8 h. This results in an annual demand of approximately 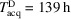 per user. Assuming that an electron microscope is operable for *H*^E^ = 5000 h per year, the maximum number of researchers it could serve at full capacity is
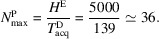
This is roughly the size of a large structural biology department in a major institute.

For a screening cryo-electron microscope with a lifespan of *L* = 10 years and an annual operable time of *H*^E^ = 5000 h, the total cost structure includes both capital and recurrent expenses. The initial acquisition cost of the microscope is 2 million euro, with an additional 250 000 euro required for facility setup and adaptation. The maintenance contract is structured with no cost for the first four years and a recurring fee of 150 000 euro per year for the remaining six years, totaling 900 000 euro. Annual consumables include 80 000 euro for items such as grids and clips, and 30 000 euro for liquid nitrogen, totaling 110 000 euro per year, or approximately 1.1 million euro over the equipment’s lifetime. Staffing requires 1.5 full-time equivalents (FTEs), with an estimated unit cost of 75 000 euro per FTE per year, resulting in 1.125 million euro across ten years. Summing all components, the total ten-year cost amounts to approximately 5.375 million euro: 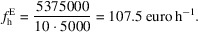


At full capacity, *N*^P^ = 36, the average waiting time is *W*_q_ = 9 h. This means that even under conservative assumptions, a single microscope can economically serve a large number of researchers. This is primarily because the instrument’s hourly cost significantly exceeds that of the researcher. Furthermore, during waiting periods, researchers may still engage in valuable activities, such as data analysis, writing or planning, so the actual productivity loss associated with waiting is typically less than its full hourly cost.

We note that any of the input quantities listed in the previous paragraph can be changed individually in the notebook included with this work, allowing them to be tailored to the specifics of each facility.

When serving at full capacity (*N*^P^ = 36 researchers), the usage time 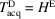 significantly exceeds the annual working hours of a single researcher (*H*^P^ = 1800 h per year), therefore, to maintain an average waiting time of less than 12 h, researchers may need to coordinate usage in shifts, including evenings and weekends, to ensure efficient access and avoid congestion.

This scheduling flexibility is common in institutes with large structural biology departments, where experienced users are trained to operate the microscope independently and can schedule their sessions around the clock to maximize instrument time.

### 3.4. High-end microscope

Consider a researcher who accesses a high-end electron microscope roughly once every three months, with a cycle time of *t*_cycle_ = 2160 h and an acquisition time of *t*_acq_ = 24 h. This leads to an annual demand of 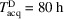 per user. Given a total microscope availability of *H*^E^ = 5000 h per year, the theoretical maximum number of users it could support is
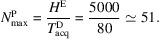


The acquisition cost of the microscope is 7.5 million euro, with an additional 250 000 euro needed for facility setup and adaptation. The maintenance contract is provided free of charge for the first four years, followed by an annual cost of 250 000 euro for the remaining six years, totaling 1.5 million euro. Consumables remain consistent with the screening microscope, at 80 000 euro for grids and clips and 30 000 euro for liquid nitrogen per year, totaling 1.1 million euro over the full period. Staffing requirements increase to two full-time equivalents (FTEs), with each FTE costing 75 000 euro annually, resulting in 1.5 million euro over the ten-year period. Additionally, the computational infrastructure (including data storage and processing capacity) will likely require a mid-life renewal, estimated at 300 000 euro. Altogether, the total ten-year cost reaches approximately 12.15 million euro. The calculation of the hourly fee for a microscope at full capacity would be 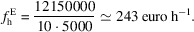


If an average waiting time of two weeks is considered acceptable, the microscope could support around *N*^P^ = 103 active researchers. Interestingly, increasing the number to *N*^P^ = 121 would raise the average waiting time to about a month; that is, the waiting time grows quite nonlinearly, which may not be an obvious *a priori* insight. However, if we put *c* = 2 microscopes to serve the *N*^P^ = 121 researchers, the waiting time drops to 17 h.

The user base can be further broadened through a structured call-for-access model. In this approach, researchers submit proposals for microscope time, and only a subset, typically those deemed most scientifically compelling, is granted access. Given the microscope’s annual capacity of approximately *H*^E^/*t*_acq_ = 208 acquisition slots, demand would far exceed availability. This competitive access mechanism is commonly employed by national facilities, synchrotrons and European research infrastructures to ensure optimal scientific return on limited high-end resources.

### 3.5. National and supranational facilities

The primary focus of this work has been to show how a rigorous, quantitative analysis of the financial and operational management of a shared research facility can predict key aspects of its working regime. In particular, by combining cost modelling with queueing theory, we demonstrate that central performance metrics, such as waiting times, can exhibit strongly nonlinear behaviour. These effects are often counterintuitive and therefore difficult to assess reliably without an explicit mathematical framework.

As stated at the outset, our approach deliberately avoids incorporating explicit strategic or political considerations at the institutional or national level. This choice is intentional: while several model parameters could in principle be adjusted to reflect strategic priorities, doing so would introduce subjective assumptions that obscure the objective relationships among measurable quantities. Our aim has instead been to isolate and rigorously analyse well defined variables, such as usage patterns, staffing levels and operating hours, and to derive results that follow directly from their mathematical treatment.

That said, extending the analysis beyond individual facilities to national and supranational infrastructure naturally introduces strategic dimensions that can profoundly shape scientific outcomes. At these scales, pooling resources and coordinating access can generate synergies that lead to substantial, and often nonlinear, gains in efficiency, expertise and scientific impact. At the European level, initiatives such as Instruct-ERIC in structural biology exemplify this model by enabling cross-border access to advanced technologies, including high-end platforms whose cost and operational complexity make them impractical to sustain independently in every country (Calzolari *et al.*, 2014 ▸; Del Bo, 2016 ▸).

This logic is well established. Early European strategic roadmaps, such as the 2008 FESP recommendations (https://www.ec-fesp.org/FESP/reports), have already emphasized that modern structural biology increasingly relies on expensive, specialized and skill-intensive instrumentation: not only cryo-electron microscopy, but also NMR spectrometers, synchrotron beamlines and the associated computational ecosystem. Organizing these resources into networks of transnational centres that provide coordinated access, training and professional support has long been recognized as an effective response to both economic and scientific constraints.

Beyond the purely economic and operational aspects captured by our model, large supranational infrastructures generate additional benefits that arise from scale and coordination. These include the formation of highly specialized technical teams, enhanced knowledge exchange across institutions, structured mentoring of new users and cumulative learning that improves long-term operational performance. The presence of multiple coordinated operators also facilitates the adoption of best practices, reduces the duplication of effort and enables more consistent and efficient use of complex facilities.

Although our framework does not explicitly model strategic considerations, it provides a natural foundation for incorporating such factors. In shared facilities, cost efficiency improves as utilization approaches capacity, yet service quality remains constrained by throughput, staffing levels and congestion. Because waiting times increase nonlinearly with demand, unstructured access quickly becomes impractical, making competitive and transparent allocation mechanisms, such as call-for-access schemes, essential to preserve timeliness and scientific productivity. Centralized and networked supra­national infrastructures are particularly well suited to implement these mechanisms, as they can sustain expert technical teams, standardize workflows, invest in automation and operate extended schedules. In this sense, European infrastructures such as Instruct-ERIC can be seen as a natural continuation of the FESP vision, combining equitable access and excellence-driven project selection with the operational efficiencies required for the sustainable operation of high-end scientific instrumentation.

## 4. Online calculator

To facilitate the practical application of the cost and queuing models described in this work, we have developed an online calculator, available at https://i2pc.es/coss/Programs/equipmentCost.html. This tool allows users to input key parameters such as the annual working hours of a researcher (*H*^P^), the hourly cost of a researcher (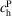), the number of researchers (*N*^P^), the annual operable hours of the equipment (*H*^E^), the equipment lifespan (*L*), the total investment over the lifespan, the time between two access requests (*t*_cycle_) and the acquisition time per usage (*t*_acq_).

The calculator computes several quantities of interest.

(i) The average number of acquisitions per researcher per year.
(ii) The total annual demand per researcher.
(iii) The hourly access fee under both single-user and multi-user scenarios.
(iv) The maximum number of researchers the equipment can support at full capacity.
(v) The average waiting time, based on an M/D/*c*/1 finite-source queue model.
(vi) The steady-state probabilities for the number of users in the system.

The user can also specify whether the cycle time is based on calendar hours (8760 h per year) or the researcher’s working hours (*H*^P^). This tool helps institutions evaluate realistic equipment-usage scenarios, financial sustainability and scheduling constraints, thereby informing decisions on procurement, facility planning and access policies.

## 5. Conclusions

This study presents a quantitative framework for assessing the cost and utilization of high-end scientific equipment, with a particular application to cryo-electron microscopes in structural biology. By combining economic modelling with queuing theory, we have derived expressions for the hourly access fee, estimated waiting times under finite user populations and identified the main factors that constrain or enable efficient operation.

Our analysis reveals that the access fee for a piece of equipment is primarily determined by its acquisition, maintenance and operational costs, amortized over its usable lifetime and effective operating hours. However, the decision on how many users an instrument can reasonably serve is governed not only by this fee, but also by the expected demand per user and the acceptable waiting time. In systems with finite user populations and irregular access patterns, queueing delays can significantly impact practical usability long before financial saturation is reached.

We have shown that under realistic assumptions, such as researchers using a cryo-electron microscope once every few weeks or months for full-day sessions, waiting times increase with the size of the user base. Although increasing the number of users reduces the per-hour access fee until the instrument reaches full capacity, it may significantly degrade service quality if waiting times become too long. This trade-off highlights the importance of modelling usage not only from an economic standpoint but also from temporal and human-resources perspectives.

A promising strategy to mitigate this trade-off is the use of automation. By streamlining session setup, minimizing manual intervention and enabling unattended or off-hours operation, automation can substantially reduce per-user acquisition time. This, in turn, improves turnover, allowing more researchers to be served without proportionally increasing waiting times. Furthermore, automation can support longer operational windows (for example, overnight use), help decouple equipment use from strict operator availability and lower the training barrier for new users. Together, these factors increase both the effective user base and the number of usable hours per year, thereby enhancing both cost efficiency and accessibility. In facilities where demand is high and skilled operators are scarce, automation becomes a crucial lever for improving throughput and sustainability.

Another critical factor in ensuring the efficiency of high-end instrumentation is the availability of well trained technical personnel. In our framework, staffing is part of the actual cost of access whenever technical support is necessary for proper operation; it is not an optional extra to be considered only if an institution happens to fund it (Sader *et al.*, 2020 ▸; Strom *et al.*, 2020 ▸; O’Toole & Marrison, 2024 ▸). This distinction is essential because the real cost of operating an instrument and the fraction of that cost recovered through user fees are conceptually different issues. Our model is designed precisely to separate these two layers: it quantifies the full access cost, including staffing, and then allows institutions or funders to decide whether that cost should be recovered fully, partially or not at all through access charges. Thus, the model supports both subsidized and fee-based regimes within the same framework, while also making the hidden consequences of understaffing visible. For example, in the case of a high-end microscope, reducing staffing by one full-time person lowers the hourly access cost by only about 6%, whereas insufficient staffing can markedly reduce usable hours through poorer operation, maintenance delays and underutilization. In practice, the modest financial savings from understaffing are often outweighed by the associated loss of throughput and scientific productivity.

These ideas are reflected in operational studies of real scientific facilities, where high utilization, skilled staffing and coordinated multi-user access are consistently identified as key determinants of performance. Industrial cryo-EM facilities, for example, rely on automation to extend operating hours and increase throughput, while trained personnel ensure reliability, optimal instrument usage and rapid problem resolution (Sader *et al.*, 2020 ▸). More broadly, analyses of shared instrumentation facilities highlight that centralization, expert staffing and structured access models are essential to sustain both efficiency and long-term operation, particularly for high-cost instruments whose maintenance and expertise requirements exceed the capacity of individual laboratories (Strom *et al.*, 2020 ▸). These studies also report practical constraints such as scheduling bottlenecks, high demand and limited availability, which closely mirror the trade-offs captured in our model. In particular, the interplay among demand, acquisition time and available support gives rise to regimes in which small changes in workload or staffing can produce disproportionately large effects on waiting times and effective capacity. Taken together, these observations provide qualitative validation of our framework, showing that the combined role of automation and staffing is not merely theoretical, but a central feature of real shared-infrastructure operation.

It is important to note that in practice, the full access fee derived from cost modelling is seldom charged to users. Access to high-end equipment is often heavily subsidized, if not entirely free, from the perspective of individual researchers. While this facilitates access and fosters scientific activity, it also obscures the actual economic burden institutions bear. Experience from shared-resource laboratories operating under full-cost-recovery frameworks shows that the real cost of access includes not only capital expenditure but also staffing, maintenance, infrastructure and day-to-day operational support, even when institutional rules or funding policies prevent these costs from being fully passed on to users through access fees (O’Toole & Marrison, 2024 ▸). In many cases, institutions therefore lack a detailed accounting of how much internal funding is implicitly allocated to support equipment usage. As a result, strategic planning and long-term sustainability may be compromised by an incomplete understanding of the actual costs involved. Making these implicit subsidies explicit, through internal cost tracking or transparent budgeting, would enhance institutional awareness and enable more informed decisions about infrastructure funding, pricing policy and equipment renewal.

While our analysis focuses on optimizing the cost-efficiency of shared scientific equipment, it is essential to acknowledge that some high-cost instruments may be acquired as part of a broader strategic vision rather than solely based on immediate user demand. Institutions or national research agencies may choose to invest in cutting-edge infrastructure to build long-term scientific capabilities, attract top talent or signal leadership in a particular field (Hallonsten, 2013 ▸; Heidler & Hallonsten, 2015 ▸). In such cases, the user base may be small initially and the equipment may not operate at full capacity. Yet, the investment can still be justified as a catalyst for research and development. These strategic acquisitions should be clearly distinguished from standard service equipment, the primary goal of which is to maximize efficiency and ensure sustainable use through well balanced user access. A clear separation between these two rationales, strategic investment and service infrastructure, helps align expectations, budgeting and operational models with each facility’s intended role.

From a strategic perspective, coordinating high-end instruments into national or supranational facilities provides both operational and financial advantages. Coordinating infrastructure reduces redundant fixed costs (such as facility adaptation and administrative overhead) and enables the formation of expert technical teams that can improve availability and throughput. Furthermore, coordinated facilities are better positioned to implement structured, competitive access models that allocate limited time to the most scientifically valuable projects, a practice already adopted in synchrotrons, national platforms and European infrastructures.

In conclusion, effective equipment planning requires a shift from intuition-based procurement to data-informed decision-making. Institutions and funding bodies must consider not only the capital and maintenance costs of scientific infrastructure but also the dynamics of user demand, access patterns and staffing logistics. The framework presented here offers a generalizable approach to designing cost-efficient, high-impact and user-sensitive equipment-sharing strategies. While our main examples focus on cryo-electron microscopy, the methods can be extended to other expensive and scarce scientific resources, contributing to more sustainable and scientifically productive research environments.
